# Extended Use of The Spanner® Temporary Prostatic Stent in Catheter-Dependent Men with Comorbidities

**DOI:** 10.1155/2022/7367851

**Published:** 2022-02-03

**Authors:** Angelo J. Cambio, Richard M. Roach, Paul Arnold, Joseph Cambio, Clifford D. Gluck, Sean P. Heron

**Affiliations:** ^1^Brown Urology Brown Physicians, West Warwick, RI, USA; ^2^Advanced Urology Institute, Oxford, FL, USA; ^3^Advanced Urology Institute, Palm Harbor, FL, USA; ^4^Carney Hospital, Dorchester, MA, USA; ^5^Pinellas Urology, St. Petersburg, FL, USA

## Abstract

**Purpose:**

This US FDA investigational device exemption (IDE) study evaluated the extended use of The Spanner® Temporary Prostatic Stent in catheter-dependent men with urinary retention who were not deemed candidates for corrective surgery but demonstrated bladder contractility.

**Materials and Methods:**

The Spanner was placed for 3 cycles of 30 days in catheter-dependent men with comorbid conditions, confirmed detrusor contractility, and catheter-associated discomfort. At each visit, postvoid residual, maximum flow rate, international prostate symptom score, quality of life, and adverse events were assessed. Voiding success was defined as PVR ≤ 150 ml at all visits.

**Results:**

One hundred seven men were enrolled at 8 US sites; 82/107 (76.6%) completed the trial, and 79/107 (73.8%) successfully maintained PVR ≤ 150 ml for the trial duration. Patients were 77.1 ± 10.6 years old; 63/107 (58.9%) were dependent on Foley and 40/107 (37.4%) on intermittent catheterization for 36.0 ± 39.3 days and 30.2 ± 45.8 days, respectively. 25/107 (23.4%) discontinuations were primarily due to voluntary patient withdrawal 9/107 (8.4%), investigator-initiated withdrawal 8/107 (7.5%), or lack of effectiveness 4/107 (3.7%). During Spanner use, the mean *Q*_max_ was 11.2 ± 6.6, mean IPSS was 7.5 ± 6.4, and mean QOL was 2.0 ± 1.6. The most prevalent device-related adverse events were asymptomatic bacteriuria 25/107 (23.4%), discomfort 10/107 (9.4%), and urinary urgency 8/107 (7.5%). No device-related serious AEs were reported.

**Conclusions:**

This study demonstrates that catheter-dependent men with sufficient bladder contractility can achieve volitional voiding and successful bladder drainage using The Spanner Temporary Prostatic Stent for extended periods of time.

## 1. Introduction

Urethral catheters are used extensively for a continuum of conditions from incontinence, bladder obstruction, retention, benign prostate hyperplasia (BPH), and bladder cancers to neurological disorders such as cerebral palsy, multiple sclerosis, and spina bifida. They provide effective passive bladder drainage for men with competent or dysfunctional bladders.

However, while the purpose of urethral catheters is to help safely manage the care and well-being of the patient, their use often leads to the opposite result. Patients with indwelling catheters report high rates of complications such as pain/discomfort, urgency/bladder spasms, blood in urine, and trauma due to catheter securement or placement, as well as restrictions in daily living and social activities [1]. Use of urinary catheters is associated with increased risk of incontinence, paraphimosis, false passage, urinary calculi, compromised kidney function, urinary tract infection (UTI), and death [2].

The prevalence and high cost of catheter-associated UTI (CAUTI) led to the creation of clinical guidelines which provide evidence-based information and recommendations for the treatment and prevention of urinary tract infections. Their effectiveness is supported by recent Centers for Disease Control and Prevention (CDC) reports showing improvement in health care-associated infection rates (HAI) [3]. The Emerging Infection Program Hospital Prevalence Survey team demonstrates significant improvements in the prevalence of hospital-associated infections (HAI) in health care settings. Fewer patients had HAI in 2015 (3.2%) than in 2011 (4.0%; *p* < 0.001) and in 2015, 10% of hospital-associated infections were UTIs and 6.1% were CAUTIs, compared with 14.4% (*p*=0.003) and 9.7% (*p*=0.005), respectively, in 2011 [4].

In spite of these successes, global sales of urinary catheters continue to grow, a fact which underscores the importance of continued implementation of the recommendations [5]. The CDC and the American Urological Association (AUA) guidelines identify temporary urethral stents as an alternative to indwelling catheters, and the CDC recommends further study of temporary urethral stenting to determine its effect on CAUTI prevention in selected patients with bladder outlet obstruction [6, 7].

Short-term urethral stenting (up to 30 days) with The Spanner® Temporary Prostatic Stent has been shown to preserve volitional voiding, improve bladder emptying, and reduce the severity of lower urinary tract symptoms with low complication rates [8]. When used subsequent to minimally invasive treatments (MIT) for BPH, The Spanner significantly increased voiding efficiency while preserving continence, with infectious adverse event rates that do not differ statistically or clinically from the MIT alone [9,10].

The successful demonstration of short-term safety and effectiveness of The Spanner led to a prospective, FDA-reviewed investigational device exemption (IDE) study of its extended sequential use (up to 90 cumulative days) in catheter-dependent men with bladder contractility refractive to BPH surgery.

## 2. Materials and Methods

### 2.1. Study Design

This IDE study was a prospective, multicenter, single-arm, open-label study to evaluate the safety and effectiveness of The Spanner in men with urinary retention who are dependent on urinary catheters. The study was overseen by Western IRB (WIRB # 20152836); each patient was given and signed an IRB-approved informed consent form prior to any study specific requirements. The ClinicalTrials.gov Identifier is NCT02643849. All authors were investigators in this study sponsored by SRS Medical.

Patients were fitted with The Spanner and underwent uroflowmetry and PVR testing at each of four visits. The stent was replaced approximately every 30 days for three cycles of use. Postvoid residual (PVR), uroflowmetry, symptom scores, quality of life scores, patient satisfaction, and adverse events were collected throughout the study [11, 12]. Cystoscopy was performed prior to the initial stent placement and after final stent removal to assess the cumulative effects of The Spanner on the urinary tract. Patients completed the study upon removal of the last stent after the 3rd month, followed by a safety telephone interview conducted two weeks later (Table 1).

### 2.2. Selection Criteria

Enrolled men were >45 years old, prescribed catheterization for urinary retention, and catheterized for <180 days with some catheter-associated pain. All patients had comorbid conditions that precluded them from pharmacologic, minimally invasive, or surgical treatment of the prostate and all had confirmed bladder contractility via urodynamic testing (detrusor contractility ≥15 cm H_2_O within 180 days of study enrollment). Men were excluded with symptomatic UTI, anatomical conditions contraindicated with the use of The Spanner as described in the Instructions For Use, penile prosthesis, known or suspected prostate cancer, pelvic irradiation, medical conditions associated with neurologic bladder, or use of anticholinergic medication [13].

### 2.3. Protocol for Use of The Spanner

**Figure 1:**
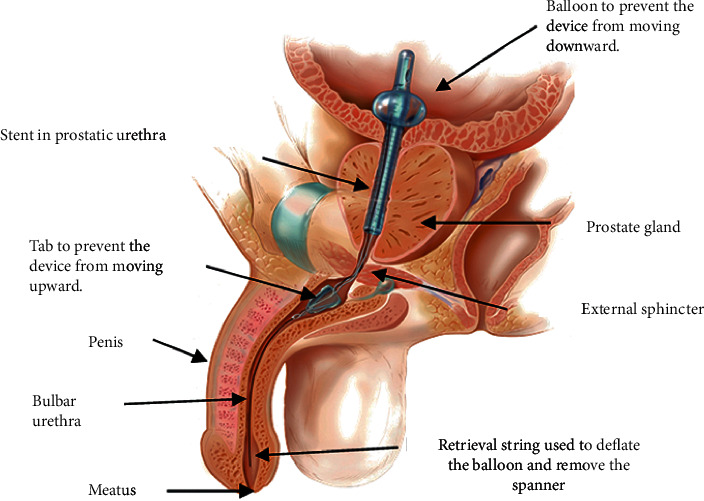
Sagittal view of The Spanner in situ (copyright SRS Medical, used with permission).

Investigators used The Spanner® Temporary Prostatic Stent and The Surveyor® (SRS Medical, North Billerica, MA, USA). The Spanner is comprised of a 20F silicone tube that holds open the prostatic urethra, deobstructing the bladder outlet without disrupting the external sphincter. The device is anchored at the bladder neck by a 5cc balloon and in the bulbar urethra by a pliable silicone distal anchor. Placement is performed by the urologist in an outpatient setting without visualization using topical anesthesia (Figure 1). Detailed descriptions of The Spanner, The Surveyor, and their use are provided elsewhere [9].

### 2.4. Definition of Infectious Complications

#### 2.4.1. Symptomatic Urinary Tract Infection (SUTI)

The protocol prespecified criteria used to define symptomatic urinary tract infection (SUTI) were based on definitions from the Center for Disease Control [14]. Study participants had to have at least ***one*** of the following signs or symptoms:

- Fever (>38°C)
- Suprapubic tenderness (with no other recognized cause)
- Costovertebral angle pain or tenderness (with no other recognized cause)
- Urinary frequency
- Urinary urgency
- Dysuria
- A urine culture with no more than two species of organisms, at least one of which is bacteria of ≥100,000 CFU/ml.

#### 2.4.2. Asymptomatic Bacteriuria

The protocol-specified criteria used to define asymptomatic bacteriuria were a urine culture of a single organism with a colony count >100,000 CFU and an asymptomatic patient.

### 2.5. Statistical Analysis

The primary objective of the study was to determine the proportion of patients who achieved and maintained adequate bladder drainage over 90 days, defined as a postvoid residual (PVR) of ≤150 ml. This objective was met if the one-sided lower bound of the 95% confidence limit for the incidence of patients who achieve adequate bladder drainage at each of the 4 evaluations (after stent placement and after each month of use) over 90 days was ≥50%. This hypothesis was tested using a one-sample exact binomial test in the intent-to-treat population (ITT) and has adequate statistical power to support the primary endpoint analysis.

Summary statistics were computed for the primary endpoint along with a 95% confidence interval on the treatment group success proportion. Secondary endpoints assessed the proportion of patients maintaining PVR volumes of ≤150 ml for 90 days and ≤250 ml over 30 and 90 days. All other analyses were conducted on the safety population. Statistical analyses used SAS® version 9.2 software (Cary, NC, USA).

## 3. Results

### 3.1. Patient Disposition

**Figure 2:**
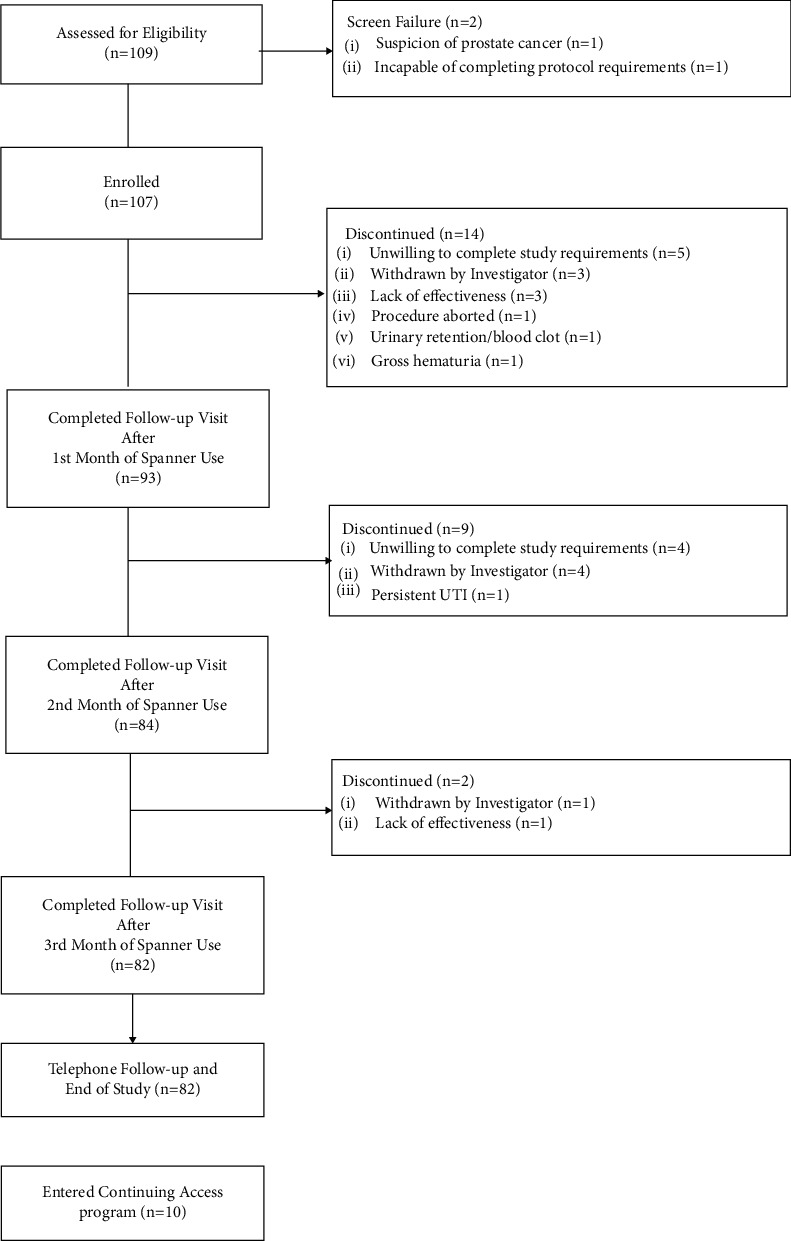
CONSORT diagram of study flow.

One hundred seven (107) catheter-dependent men with comorbid conditions which precluded them from pharmaceutical, minimally invasive, or surgical methods of treating prostatic obstruction were enrolled from August 3^rd^, 2016, until October 25^th^, 2018. Eighty-two men (82/107; 76.6%) completed the study, and 25/107 (23.4%) discontinued; primarily due to patient unwillingness to complete study requirements (9/107; 8.4%), physician-mediated withdrawal based on the determination that the patient was unable to complete study requirements (8/107; 7.5%), and lack of effectiveness (4/107; 3.7%). After completion of the study, ten patients entered the Continuing Access Program, where additional stents were placed for 30 day cycles (CONSORT study diagram, Figure 2).

### 3.2. Baseline Characteristics

The study enrolled an aged population with significant comorbidities and compromised bladder health (Table 2). The average patient age was 77.1 ± 10.6 years. A Foley catheter was used by 63/107 (58.9%) subjects and clean intermittent catheterization (CIC) was used by 40/107 (37.4%). Two subjects reported using both Foley catheter and CIC prior to first treatment. Six subjects 6/107 (5.6%) were diagnosed to be in urinary retention and were prescribed catheterization; however, the Investigator did not confirm catheter usage at the time of screening; they are categorized as “Unconfirmed catheter type.”

The average duration of Foley catheter use prior to enrollment was 36.0 ± 39.3 days and intermittent catherization was 30.2 ± 45.8 days. Baseline incidence of trabeculation, cystitis, and diverticulum was 64/107 (60.0%), 11/107 (10.3%), and 22/107 (20.6%).

### 3.3. Device Use

There were 284 stent insertions with procedure time averaging 15.8 ± 9.6 minutes. Most of the time (268/284; 94.4%) the investigators rated the insertion easy or very easy and most patients (260/284; 91.6%) reported mild pain or no pain. The average duration of stent use was 88.5 ± 8.7 days for the patients who completed all 3 cycles and 25.6 ± 19.3 days for the discontinued patients. In total, patients had The Spanner in situ for 7,897 days. The stent size distribution was as follows: size 4 (0%), size 5 (1.9%), size 6 (12.5%), size 7 (38.6%), size 8 (30.1%), and size 9 (16.9%).

### 3.4. Study Endpoints

Seventy-nine (79) of the 107 (73.8%, [0.644,0.819 95% CI] *p* < 0.0001) enrolled men met the primary study endpoint, demonstrating adequate bladder drainage of PVR ≤ 150 ml during three months of sequential use (ITT). The secondary endpoints demonstrated that, after 30 days, 87/107 (81.3%) patients had a PVR ≤ 150 ml, 88/107 (82.3%) had a PVR ≤ 250 ml, and after 90 days, 81/107 (75.7%) of patients maintained a PVR ≤ 150 ml (ITT). Subgroup analyses of age, catheter type, and comorbidities at enrollment demonstrated consistent treatment effects across subgroups (Table 3).

### 3.5. Urinary Outcomes

PVR, *Q*_max_, IPSS, and QOL measurements conducted during the study demonstrate successful voiding. Most patients were able to maintain effective bladder emptying (PVR ≤ 150 ml) with stable PVR, *Q*_max_, IPSS, and QOL (Table 4).

### 3.6. Pain and Satisfaction Patient-Reported Outcomes

At each visit, fewer than 25% of patients reported Spanner-related pain, and after 3 months of use, of the 16/82 (19.5%) who reported pain, 8/16 (50%) reported pain occurred less than once per day (Table 5).

Patients rated their satisfaction with the stent at each follow-up visit. After 3 months of use, the majority of the patients reported being very satisfied or satisfied with The Spanner stent 67/82 (81.7%) and would recommend The Spanner to another man 77/82 (93.9%).

As men enrolled in this study had experienced both urinary catheterization and the stent, they were asked to compare the devices. After 3 consecutive months of Spanner use, 57/63 (90.5%) and 55/60 (91.7%) of patients reported that they preferred or greatly preferred Spanner compared to Foley or intermittent catheterization, respectively.

### 3.7. Cystoscopy

Spanner use had negligible impact on the bladder or urethra based on cystoscopic visualization. After 3 months of use, incidence of trabeculation, cystitis, and diverticulum was 47/82 (57.32%), 9/82 (10.98%), and 16/82 (19.51%), virtually identical to baseline results (Table 2).

### 3.8. Continued Access Protocol

Ten patients entered the Continued Access Protocol (CAP) upon completion of the 3 cycles and had The Spanner replaced approximately every 30 days. The patients attended 64 visits for 1–15 months during this program.  Table 6 depicts the number of patients by PVR category for the first 8 months of the program. Only one patient continued in CAP beyond 8 months; he was tracked for 15 months with a PVR ≤ 150 ml at each visit.

### 3.9. Adverse Events

Patients reported 173 adverse events in the study; the investigators determined that 101/173 (58.4%) were related to the device or the procedure (Table 7). Each AE related to the device or the procedure was a Grade I or Grade II on the Clavien–Dindo classification system. Most AEs were mild (151/173; 87.3%) to moderate (20/173; 11.6%) in severity. Type and frequency of adverse events were as expected based on previous studies and were not exacerbated in this compromised patient population. Four (4/107; 3.7%) patients experienced 6 SUTI events during the course of the study; for all events, The Spanner had been in place for >2 days and was present on the day of the start date of the event. The SUTI rate was 0.76/1,000 days. Fifteen of the 107 patients (14.0%) reported 16 SAEs, all of which recovered, none of which were related to the procedure or the device, and 9 of 16 (56.3%) were associated with preexisting conditions.

Type and frequency of adverse events were not noticeably affected by continued use of The Spanner in the CAP program. One patient experienced 2 SUTI events during the course of the CAP program and no new AE types were observed (Table 8).

## 4. Discussion

The implementation of the recommendations of the CDC and other national health organizations has improved the prevalence of health care-associated infections in the US. However, continued efforts are still needed as size of the urinary catheter market continues to grow.

In the 2009 Guideline for Prevention of Catheter-Associated Urinary Tract Infections^7^, the CDC recommends limiting urinary catheter use for the appropriate indications and leaving them in place only as long as needed. Question 1 A in this guideline indicates that further research is needed to document the benefits of using temporary urethral stenting as an alternative to indwelling urethral catheterization in selected patients with bladder outlet obstruction. Our findings describe the benefits of temporary urethral stenting in a catheter-dependent population.

The temporary urethral stent, The Spanner, has been marketed since 2006 as a short-term (up to 30 days) alternative to urinary catheterization following MIT. This IDE study evaluates the safety and effectiveness of The Spanner in catheter-dependent men. It provides long-term evidence (up to 90 days) that replacing a passive draining catheter with successive use of The Spanner results in (a) low PVR volumes (median PVR = 30 ml), (b) LUTS of mild severity (median IPSS = 7.3), (c) improved quality of life, as men were mostly satisfied with their current urinary condition (median QOL = 2.0), and (d) low infectious complication rates; SUTI (3.7%; 0.76/1,000 days) and asymptomatic bacteriuria (23.4%; 3.67/1,000 days). Of those who completed the trial, 68% underwent a BPH surgery within six months.

The low infectious complication rates were based on device use for up to 90 days. In comparison, the colonization rate for men catheterized for this length of time would be approximately 100% [15]. Saint et al. report that 98% of weekly urine specimens from chronically catheterized patients contained >105 CFU/ml bacteria and Stark et al. demonstrate that, in the presence of an indwelling urethral catheter, the rate of acquisition of high-level bacteriuria is approximately 5% per day [16, 17].

These complication rates are congruent with earlier studies of extended use of The Spanner. Abdul-Muhsin et al. of The Mayo Clinic report using The Spanner in 33 BPH patients who were refractory to medical management. The stents were replaced every 4–6 weeks with up to 14 stent exchanges. They found no bacterial colonization or infection when the stent was removed within 20 days of placement; the investigators concluded that the symptomatic infection rate with the first utilization of Spanner was rare [18].

Roach treated 32 male retention patients with underlying comorbidities with multiple [2–18] Spanner stents. SUTI occurred in 2.5% of placements (0.68/1,000 days). He contrasts this with the CDC estimated CAUTI rate of 3.1 to 7.5/1,000 days experienced in US acute care hospitals [19].

Sabharwal et al. replaced indwelling catheters with 5 cycles of The Spanner in 22 chronically catheterized men with the goal of reducing bacterial colonization rate. He postulated that the resumption of natural filling and emptying and the lack of external device components would allow the body to naturally protect against bacterial colonization. All patients were colonized at enrollment, 59.1% at the first stent exchange, 13.6% at the second exchange, and 4.5% at each of the subsequent 3 exchanges. He suggests that replacing an indwelling catheter with The Spanner may interrupt the cycle of bacterial colonization in the urinary tract [20].

This study was limited to men with detrusor contractility confirmed via pressure-flow test, a difficult test to conduct in this compromised population. Overall these studies are of sufficient quality to provide firm conclusions regarding the bladder emptying and infection rates associated with the extended use of The Spanner in catheter-dependent men and should provide the CDC with appropriate evidence to consider in the guidance document Prevention of Catheter-Associated Urinary Tract Infections [21].

## 5. Conclusions

This IDE study of The Spanner demonstrates that extended use of The Spanner facilitates volitional voiding and provides bladder drainage, low infection rates, low IPSS symptom scores, and high QOL in men in urinary retention.

## Figures and Tables

**Table 1:** Study activities.

Activity	Screening, catheter cessation, enrollment, 1st Spanner placement	Follow-up visit after 1^st^ month of Spanner use	Follow-up visit after 2^nd^ month of Spanner use	Follow-up visit after 3^rd^ month of Spanner use	Follow-up phone call
Informed consent	●				
Medical history^i^, physical, digital rectal exam	●				
Concomitant medication assessment	●	●	●	●	
Urinalysis	●	●	●	●	
Catheterization assessment	●				●
Cystoscopy	●			●	
Eligibility assessment	●				
Study enrollment	●				
Surveyor measurement	●				
Stent placement	●	●	●		
Stent removal		●	●	●	
Adverse event assessment^i, ii^	●	●	●	●	●
Uroflowmetry and post void residual	●	●	●	●	
Satisfaction questionnaire	●	●	●	●	
Serum creatinine	●	●	●	●	
International prostate symptom score including quality of life^iii^		●	●	●	
End of study					●

List of study activities conducted at each visit. Visit 1 included consent, assessment of selection criteria, enrollment, placement of first stent, and measurement of PVR with The Spanner in situ. The Spanner was exchanged at each of the next three study visits for a total of 90 days of stent use. Study ended after the final follow-up phone call to assess patient safety. (i) Medical History coded to MedDRA System Organ Class. AEs and SAEs were coded to preferred terms. (ii) Symptomatic UTI definition based on the current CDC guidance. Urinary Tract Infection (Catheter-Associated Urinary Tract Infection (CAUTI) and Non-Catheter-Associated Urinary Tract Infection (UTI)) and Other Urinary System Infection (USI)) Events, January 2015. (iii) Symptom Assessment based on AUA Symptom Score/IPSS as published by Barry et al. [11] and by Cockett et al. [12].

**Table 2:** Characteristics at enrollment.

	Mean ± SD, median, Min-Max, *n*
*Demographics*	
Age (years)	77.1 ± 10.6, 78, 50–97, 107
BMI	27.6 ± 5.5, 27, 19–50, 102

*Catheter type*	*n*/*N* (%)
Foley^i^	63/107 (58.9%)
Intermittent^i^	40/107 (37.4%)
Unconfirmed catheter type	6/107 (5.6%)

*Bladder health*	
Trabeculation	64/107 (60.0%)
Cystitis	11/107 (10.3%)
Diverticulum	22/107 (20.6%)

*Medical history*	
Genitourinary other than urinary retention	90/107 (84.1%)
Cardiovascular	88/107 (82.2%)
Endocrine/metabolic	69/107 (64.5%)
Medical and surgical procedures	50/107 (46.7%)
Gastrointestinal	41/107 (38.3%)
Musculoskeletal	30/107 (28.0%)
Psychiatric	28/107 (26.2%)
Neurological	18/107 (16.8%)
Respiratory	18/107 (16.8%)
HEENT (head, eyes, ears, nose, and throat)	14/107 (13.1%)
Hematologic/lymphatic	14/107 (13.1%)
Allergic/immunologic	9/107 (8.4%)
Dermatological	4/107 (3.7%)
Substance abuse	4/107 (3.7%)
General	1/107 (0.9%)

Baseline data for all enrolled patients including medical history categorized by Charlson Comorbidity Index. ^i^Two patients reported both Foley and intermittent catheter use and are included in both groups.

**Table 3:** Subgroup analyses.

** **	Age group		Catheter type		Comorbidities				Overall study data
	Age	Age	Foley	CIC	MI	CHF	DM	PVD	
	50–77	78–97							
Number and % of subjects in subgroup									
*n*	50 (46.7%)	57 (53.3%)	63 (58.9%)	40 (37.4%)	40 (37.4%)	34 (31.8%)	28 (26.2%)	25 (23.4%)	107
Met primary endpoint-adequate bladder drainage									
*n*/*N*	36/50	44/57	47/63	30/40	28/40	27/34	23/28	18/25	79/107
(%)	72.0%	77.2%	74.6%	75.0%	70.0%	79.4%	82.1%	72.0%	73.8%
Contracted SUTI									
*n*/*N*	3/50	1/57	3/63	1/40	3/40	0/34	2/28	0/25	4/107
(%)	6.0%	1.7%	4.7%	2.5%	7.5%	0.0%	7.1%	0.0%	3.7%

Subgroup analysis data for the two primary endpoint treatment effects: (a) adequate bladder drainage, and (b) SUTI. Subgroup analysis by (i) age (segregated by median age), (ii) catheterization use at enrollment (foley; clean intermittent catheterization (CIC)), and (iii) most prevalent comorbidities at enrollment (myocardial infarction (MI), congestive heart disease (CHF), diabetes mellitus [DM], peripheral vascular disease (PVD)).

**Table 4:** PVR, *Q*_max_, IPSS, and QOL measured during Spanner use.

Characteristics	1st Spanner placement	1^st^ month of Spanner use	2^nd^ month of Spanner use	3^rd^ month of Spanner use	Total
PVR^i^					
Mean ± SD	65.6 ± 107.1	45.4 ± 43.4	46.7 ± 78.9	53.5 ± 66.4	53.3 ± 78.9
Median	35.0	30	27	36	31
Min-Max	0–856.8	0–176.0	0–547.1	0–537.0	0–856.8
*n*	101	88	84	82	355

*Q*_max_ (ml/sec)					
Mean ± SD	11.9 ± 7.0	11.4 ± 7.1	11.8 ± 6.4	9.6 ± 5.4	11.2 ± 6.6
Median	10.0	10.1	11.6	8.2	10
Min-Max	1.0–34.0	1.6–38.4	0.9–33.2	0.8–27.0	0.8–38.4
*n*	93	84	73	73	323

IPSS	NA^ii^				
Mean ± SD		7.7 ± 6.8	7.6 ± 6.2	7.1 ± 6.2	7.5 ± 6.4
Median		5	6	5	5
Min-Max		0–35	0–29	0–29	0–35
*n*		89	82	82	253

QOL	NA^ii^				
Mean ± SD		2.0 ± 1.6	2.0 ± 1.5	2.0 ± 1.7	2.0 ± 1.6
Median		2	2	1	2
Min-Max		0–6	0–6	0–6	0–6
*n*		89	82	82	253

Standard urological measures as assessed at each study visit for all enrolled patients. (i) Patients with a PVR > 350 ml were removed from the study. Patients with a PVR > 250 ml and <350 ml were scheduled for a follow-up visit within one week to monitor PVR. (ii) Patients were incapable of voluntary voiding at enrollment so the IPSS, a questionnaire assessing symptoms occurring during voiding, was not administered.

**Table 5:** Pain assessment.

** **	1^st^ month of Spanner use	2^nd^ month of Spanner use	3^rd^ month of Spanner use
	*n*/*N* (%)	*n*/*N* (%)	*n*/*N* (%)
Pain with urological device (catheter or Spanner) over last 30 days			
** **	21/93 (22.6%)	20/84 (23.8%)	16/82 (19.5%)

*Frequency of pain*			
Less than once per week	7/21 (33.3%)	6/20 (30.0%)	5/16 (31.3%)
Several times per week	2/21 (9.5%)	4/20 (20.0%)	3/16 (18.8%)
At least once per day	5/21 (23.8%)	3/20 (15.0%)	2/16 (12.5%)
At least twice per day	4/21 (19.1%)	2/20 (10.0%)	5/16 (31.3%)
All of the time	3/21 (14.3%)	5/20 (25.0%)	1/16 (6.3%)

*Pain level (1–5 scale)*			
Mean ± SD	2.10 ± 1.51	2.00 ± 1.38	2.50 ± 1.21
Median	1.0	1.0	3.0
Min-Max	1–5	1–5	1–5

Pain frequency and severity assessed at each study visit for all enrolled patients. Catheter-related pain was assessed at the first visit, prior to insertion of The Spanner. Spanner-related pain was assessed at each subsequent study visit, after 30 days of use.

**Table 6:** PVR summary for patients in the Continuing Access Program.

PVR category	*n* (%)							
	Visit 1 (*n* = 10)	Visit 2 (*n* = 9)	Visit 3 (*n* = 9)	Visit 4 (*n* = 7)	Visit 5 (*n* = 6)	Visit 6 (*n* = 5)	Visit 7 (*n* = 5)	Visit 8 (*n* = 2)
PVR ≤ 150 ml	10 (100%)	8 (89%)	7 (78%)	6 (86%)	5 (83%)	4 (80%)	4 (80%)	1 (50%)
PVR 151–250 ml	0%	1 (11%)	1 (11%)	1 (14%)	1 (17%)	1 (20%)	0%	0%
PVR > 250 ml	0%	0%	1 (11%)	0%	0%	0%	1 (20%)	1 (50%)

Stratification of all enrolled CAP patients by PVR volume category with The Spanner in situ.

**Table 7:** Adverse events related to the procedure/device.

Adverse events^i^	*n*/*N* (%)	Events
Asymptomatic bacteriuria	25/107 (23.36%)	29
Pain	10/107 (9.35%)	10
Urinary urgency	8/107 (7.48%)	8
Urinary frequency	6/107 (5.61%)	6
Dysuria	6/107 (5.61%)	6
Voiding difficulty	6/107 (5.61%)	6
Hematuria	5/107 (4.67%)	5
Urinary incontinence	4/107 (3.74%)	5
Urinary retention	4/107 (3.74%)	5
Symptomatic urinary tract infection^ii^	4/107 (3.74%)	6
Penile pain	3/107 (2.80%)	3
Residual urine	3/107 (2.80%)	3

Display of adverse events reported by 2% or more of all enrolled patients by MedDRA preferred code. (i) The following AEs were reported by less than 2% of the patients: urinalysis abnormal (2/107; 1.9%), bladder discomfort (1/107; 0.9%), calculus urinary bladder (1/107; 0.9%), cloudy urine (1/107; 0.9%), nocturia (1/107; 0.9%), painful erection (1/107; 0.9%), postvoid dribbling (1/107; 0.9%), and pus cells in urine (1/107; 0.9%). (ii) Symptomatic UTI definition based on then current CDC guidance. Urinary Tract Infection (Catheter-Associated Urinary Tract Infection (CAUTI) and Non-Catheter-Associated Urinary Tract Infection (UTI)) and Other Urinary System Infection (USI)) Events, January 2015.

**Table 8:** Procedure and/or device-related adverse events for patients in the Continuing Access Program.

CAP AE	*n*/*N* (%)	Events
Bacteriuria	2/12 (16.67%)	5
Hematuria	1/12 (8.33%)	1
Urinalysis abnormal	1/12 (8.33%)	3
Residual urine	1/12 (8.33%)	1
Urinary retention	1/12 (8.33%)	1
Symptomatic urinary tract infection^i^	1/12 (8.33%)	2

Display of all adverse events reported by all patients enrolled in CAP by MedDRA preferred code. (i) Symptomatic UTI definition based on then current CDC guidance. Urinary Tract Infection (Catheter-Associated Urinary Tract Infection (CAUTI) and Non-Catheter-Associated Urinary Tract Infection (UTI)) and Other Urinary System Infection (USI)) Events, January 2015.

## Data Availability

The datasets collected and/or analyzed during the current study are available from the corresponding author on reasonable request and upon IRB approval.
